# An internally and externally validated nomogram for predicting the risk of irinotecan-induced severe neutropenia in advanced colorectal cancer patients

**DOI:** 10.1038/bjc.2015.122

**Published:** 2015-04-16

**Authors:** W Ichikawa, K Uehara, K Minamimura, C Tanaka, Y Takii, H Miyauchi, S Sadahiro, K Fujita, T Moriwaki, M Nakamura, T Takahashi, A Tsuji, K Shinozaki, S Morita, Y Ando, Y Okutani, M Sugihara, T Sugiyama, Y Ohashi, Y Sakata

**Affiliations:** 1 Division of Medical Oncology, Department of Medicine, Showa University, School of Medicine, 1-5-8 Hatanodai, Shinagawa-ku, Tokyo 142-8555, Japan; 2 Division of Surgical Oncology, Department of Surgery, Nagoya University Graduate School of Medicine, 65 Tsurumai-cho, Showa-ku, Nagoya 466-8560, Japan; 3 Department of Surgery, Mitsui Memorial Hospital, Kanda-Izumi-cho 1, Chiyoda-ku, Tokyo 101-8643, Japan; 4 Department of Surgery, Gifu Prefectural General Medical Centre, 4-6-1 Noishiki, Gifu 500-8717, Japan; 5 Department of Surgery, Niigata Cancer Centre Hospital, 2-15-3 Kawagishi-cho, Chuo-ku, Niigata 951-8566, Japan; 6 Department of Frontier Surgery, Graduate School of Medicine, Chiba University, 1-8-1 Inohana, Chuo-ku, Chiba 260-8670, Japan; 7 Department of Surgery, Tokai University, 143 Shimoyasuya, Isehara 259-1193, Japan; 8 Institute of Molecular Oncology, Showa University, 1-5-8 Hatanodai, Shinagawa-ku, Tokyo 142-8555, Japan; 9 Division of Gastroenterology, University of Tsukuba, 1-1-1 Tennodai, Tsukuba 305-8575, Japan; 10 Comprehensive Cancer Centre, Aizawa Hospital, 2-5-1 Honjo, Matsumoto 390-8510, Japan; 11 Department of Medical Oncology, Kobe City Medical Centre General Hospital, 2-1-1 Minatojimaminamimachi, Chuo-ku, Kobe 650-0047, Japan; 12 Division of Clinical Oncology, Hiroshima Prefectural Hospital, 1-5-54 Ujina-Kanda, Minami-ku, Hiroshima 734-8530, Japan; 13 Department of Biomedical Statistics and Bioinformatics, Kyoto University Graduate School of Medicine, Yoshida-Konoe-cho, Sakyo-ku, Kyoto 606-8501, Japan; 14 Department of Clinical Oncology and Chemotherapy, Nagoya University Hospital, 65 Tsurumai-cho, Showa-ku, Nagoya 466-8560, Japan; 15 Medical Affairs Department, Daiichi Sankyo, 3-5-1 Nihonbashi-Honcho, Chuo-ku 103-8426, Tokyo, Japan; 16 Clinical Data & Biostatistics Department, Daiichi Sankyo, 1-2-58 Hiromachi, Shinagawa-ku, Tokyo 140-8710, Japan; 17 Department of Obstetrics and Gynaecology, Iwate Medical University School of Medicine, 19-1 Uchimaru, Morioka 020-8505, Japan; 18 Department of Integrated Science and Engineering for Sustainable Society, Faculty of Science and Engineering, Chuo University, 1-13-27 Kasuga, Tokyo 112-8551, Japan; 19 CEO, Misawa City Hospital, 164-65, Aza Horiguchi, Oaza Misawa, Misawa, Aomori 033-0022, Japan

**Keywords:** colorectal cancer, irinotecan, UGT1A1, neutropenia, nomogram

## Abstract

**Background:**

In Asians, the risk of irinotecan-induced severe toxicities is related in part to UGT1A1*6 (UGT, UDP glucuronosyltransferase) and UGT1A1*28, variant alleles that reduce the elimination of SN-38, the active metabolite of irinotecan. We prospectively studied the relation between the *UGT1A1* genotype and the safety of irinotecan-based regimens in Japanese patients with advanced colorectal cancer, and then constructed a nomogram for predicting the risk of severe neutropenia in the first treatment cycle.

**Methods:**

Safety data were obtained from 1312 patients monitored during the first 3 cycles of irinotecan-based regimen in a prospective observational study. In development of the nomogram, multivariable logistic regression analysis was used to test the associations of candidate factors to severe neutropenia in the first cycle. The final nomogram based on the results of multivariable analysis was constructed and validated internally using a bootstrapping technique and externally in an independent data set (*n*=350).

**Results:**

The *UGT1A1* genotype was confirmed to be associated with increased risks of irinotecan-induced grade 3 or 4 neutropenia and diarrhoea. The final nomogram included type of regimen, administered dose of irinotecan, gender, age, *UGT1A1* genotype, Eastern Cooperative Oncology Group performance status, pre-treatment absolute neutrophil count, and total bilirubin level. The model was validated both internally (bootstrap-adjusted concordance index, 0.69) and externally (concordance index, 0.70).

**Conclusions:**

Our nomogram can be used before treatment to accurately predict the probability of irinotecan-induced severe neutropenia in the first cycle of therapy. Additional studies should evaluate the effect of nomogram-guided dosing on efficacy in patients receiving irinotecan.

Irinotecan is an effective drug for advanced colorectal cancer, as a single agent (Shimada *et al*, 1993) or in combination with a fluoropyrimidine (Goto *et al*, 2006; Muro *et al*, 2010; Komatsu *et al*, 2011), with or without a monoclonal antibody (Saltz, 2013). Irinotecan is a pro-drug, and its active metabolite SN-38 has both antitumour activities and toxicities (Senter *et al*, 2001; Xu *et al*, 2002). SN-38 is inactivated into SN-38 glucuronide (SN-38G) mainly by UDP-glucuronosyltransferase 1A1 (UGT1A1) (Iyer *et al*, 1998).

Genetic polymorphisms in *UGT1A1,* such as UGT1A1*28 in Caucasians and Asians and UGT1A1*6 only in Asians, contribute to interpatient variability in the pharmacokinetics and toxicities of irinotecan, particularly severe neutropenia (Ando *et al*, 2000; Innocenti *et al*, 2004; Minami *et al*, 2007; Innocenti *et al*, 2009; Chen *et al*, 2014). In 2005, the US Food and Drug Administration recommended that the package insert of irinotecan be amended to encourage the use of a reduced starting dose in patients homozygous for UGT1A1*28 (*28/*28). In 2008, the Ministry of Health, Labour, and Welfare of Japan likewise recommended that the package insert be revised to warn of the risk of severe irinotecan-related neutropenia in Japanese patients who are either homozygous for UGT1A1*6 or UGT1A1*28 or heterozygous for both UGT1A1*6 and UGT1A1*28. Subsequently, diagnostic genotyping for the UGT1A1*6 and UGT1A1*28 was approved in Japan and covered by health insurance.

However, factors other than the *UGT1A1* genotype may contribute to irinotecan-induced severe toxicity such as neutropenia and diarrhoea. Other non-genetic factors, such as organ functions, age, gender, co-morbidities, and performance status (PS), should therefore be comprehensively considered in predicting the risk of severe irinotecan-related toxicities (Innocenti *et al*, 2004; Marcuello *et al*, 2004; Rouits *et al*, 2004; Innocenti and Ratain, 2006; Kweekel *et al*, 2008; Liu *et al*, 2008).

We designed a prospective observational study to evaluate the effects of *UGT1A1* genotypes and non-genetic factors on the efficacy and safety of irinotecan-based regimens in Japanese patients with advanced colorectal cancer. Our primary objective was to demonstrate non-inferiority of the response to irinotecan-based regimens in terms of progression-free survival between patients harbouring UGT1A1*6 or UGT1A1*28 and patients without these polymorphisms. The secondary objective was to evaluate the relation between *UGT1A1* genotype and the safety of irinotecan-based regimens. The results of the final analysis of outcomes will be available in 2015; however, we now report the results of a planned interim analysis of safety data from 1312 patients and describe the development of a nomogram for predicting the risk of irinotecan-induced severe neutropenia, with external validation using an independent cohort of 350 patients.

## Materials and methods

### Study design and patient eligibility

This multicentre, open-label, prospective, non-interventional, observational study was conducted at 299 sites in Japan. Eligible patients had to (1) have a histologically confirmed diagnosis of adenocarcinoma of the colon or rectum, (2) have advanced or metastatic disease that was not amenable to curative resection, (3) undergo genotyping for UGT1A1*1, *6 and *28 before treatment, and (4) be scheduled to receive FOLFIRI (Muro *et al*, 2010) (folinic acid (200 mg m^−^^2^) and irinotecan (150 mg m^−^^2^) and then a bolus injection of 5-fluorouracil (400 mg m^−^^2^) on day 1 and a continuous infusion of 5-fluorouracil (2400 mg m^−^^2^) over 46 h, repeated every 2 weeks), IRIS (Muro *et al*, 2010; Komatsu *et al*, 2011) (irinotecan (125 mg m^−^^2^) on days 1 and 15 and S-1 (40–60 mg according to body surface area) twice daily for 2 weeks, repeated every 4 weeks), SIR (Goto *et al*, 2006) (irinotecan (150 mg m^−^^2^) on day 1 and S-1 twice daily for 2 weeks, repeated every 3 weeks), or bi-weekly irinotecan monotherapy (150 mg m^−^^2^), with or without molecular targeted agents and irrespective of the treatment line. The recommended dose of irinotecan for FOLFIRI is 150 mg m^−2^ for Japanese, which is less than the 180 mg m^−2^ dose recommended for Caucasians, and the recommended treatment schedule in Japan (150 mg m^−2^, on day 1, repeated every 2 weeks) differs from that in Europe and the US (350 mg m^−2^, on day 1, repeated every 3 weeks). Eligible patients also had to have adequate bone marrow reserve and liver and renal functions and provide written informed consent before enrolment. Patients were excluded if they had conditions precluding the use of irinotecan-based regimens, an Eastern Cooperative Oncology Group (ECOG) PS of 3 or 4, or a history of pelvic irradiation. The protocol was reviewed and approved by an independent ethics committee or the institutional review board of each participating centre. The study was conducted in accordance with the Declaration of Helsinki and local ethical and legal requirements. This observational study was registered with the website http://www.ClinicalTrials.gov (reference identification: NCT 01039506) and the website http://www.clinicaltrials.jp/ (reference identification: JapicCTI-090945).

### Treatment and evaluation of safety

The choice of treatment regimen and dosage was left to the investigator's discretion. As described previously, 4 weeks of treatment was considered 1 cycle for FOLFIRI (Muro *et al*, 2010), IRIS (Muro *et al*, 2010; Komatsu *et al*, 2011), or irinotecan monotherapy (Shimada *et al*, 1993), and 3 weeks of treatment was considered 1 cycle for SIR (Goto *et al*, 2006). Relative dose-intensity was defined as the ratio of the cumulative administered dose to the scheduled total dose for the first 3 cycles of treatment (Shimada *et al*, 1993; Goto *et al*, 2006; Muro *et al*, 2010; Komatsu *et al*, 2011). The *UGT1A1* genetic profiles were categorised into three groups as described previously (Sato *et al*, 2011): wild-type (*1/*1), heterozygous (*1/*6, *1/*28), and homozygous (*6/*6, *6/*28, *28/*28). Clinical findings and laboratory tests were evaluated every 2 weeks during the first 3 cycles. (Evaluations at the times of drug administration were mandatory.) After the first 3 cycles, evaluations for safety were performed according to standard clinical practice. Baseline data at registration and detailed toxicities during the first 3 cycles were prospectively recorded via a web-based electronic data collection system. The types and severities of adverse events were graded according to the National Cancer Institute's Common Terminology Criteria for Adverse Events (NCI-CTCAE), version 3.0, and the highest grade of each adverse event was recorded. Safety analyses focused on neutropenia and diarrhoea because these are the most common dose-limiting toxicities associated with irinotecan-based regimens (Ando *et al*, 2000; Innocenti *et al*, 2004; Kweekel *et al*, 2008).

### Statistical analysis

The relative risk (RR) of grade 3 or 4 neutropenia or diarrhoea in the heterozygous or homozygous groups was calculated as compared with that in the wild-type group. Fisher's exact test was used for group comparisons.

### Nomogram model building and validation

Multivariable logistic regression analysis was performed to examine the relations of various factors to the occurrence of severe neutropenia of grade 3 or 4 in the first cycle. The multivariable logistic regression model included the following factors: treatment line (first, second, or later line), regimen (FOLFIRI, irinotecan+S-1, or irinotecan monotherapy), administered dose of irinotecan, gender, age, *UGT1A1* genotype (wild-type, heterozygous, or homozygous), ECOG PS (0, 1, or 2), molecular targeted agents, prior surgery, prior radiation, pre-treatment absolute neutrophil count, and pre-treatment total bilirubin level, based on previous reports (Innocenti *et al*, 2004; Marcuello *et al*, 2004; Rouits *et al*, 2004; Innocenti and Ratain, 2006; Hoskins *et al*, 2007; Kweekel *et al*, 2008; Liu *et al*, 2008; McLeod *et al*, 2010; Shiozawa *et al*, 2013). Missing values for the pre-treatment absolute neutrophil count (*n*=68, 5.2%) and total bilirubin levels (*n*=127, 9.7%) were imputed by median imputation (Little and Rubin, 2002) for each *UGT1A1* genotype. All categorical predictors were modelled using dummy variables, while all continuous predictors were modelled using restricted quadratic splines (Greenland, 1995) based on 2 knots for the tertiles to relax linearity assumptions. The final model was chosen on the basis of variables that had *P*<0.10 on a backward step-down selection process.

Nomogram validation consisted of discrimination and calibration. Discrimination refers to a nomogram model's ability to correctly distinguish two classes of outcomes. First, for internal validation, we used both a bootstrap method with 1000 resamples and a 10-fold cross-validation with 200 repetitions to estimate the bias-corrected or over-fitting corrected predictive accuracy of the model, which is expressed as the concordance index (c-index). Second, we assessed calibration, which compares the predicted probability with the observed outcome in 10 groups partitioned by the decile of the predicted probabilities.

External validation was performed by applying the prediction model to an independent cohort of 350 patients with advanced colorectal cancer who met the same eligibility criteria as the original cohort and were from six independent sites in Japan. The protocol for external validation was also reviewed and approved in each institution. Discriminative power and calibration in the independent cohort were also evaluated.

SAS version 9.2 (SAS Institute, Cary, NC, USA) was used to perform all statistical analyses. All *P* values were two-sided, and *P* values of<0.05 were considered to indicate statistical significance.

## Results

### Patient and treatment characteristics

Between October 2009 and March 2012, a total of 1376 patients were enrolled. Sixty-four patients were excluded for the following reasons: 42 had no case report forms submitted by the investigators; 15 did not receive irinotecan-based regimens; 4 patients did not meet the inclusion criteria; and 3 withdrew consent after registration. Data from the remaining 1312 patients were included in safety analysis and nomogram development. The baseline characteristics are summarised in Table 1. The *UGT1A1* genotype was wild-type in 47.9% of the patients, heterozygous in 41.1%, and homozygous in 11.1%. Nearly 80% of the patients received irinotecan-based regimens as second- or later-line chemotherapy. The rate of received regimen type was similar among the three groups according to the *UGT1A1* genotype. Similar to a previous study (Sai *et al*, 2004; Liu *et al*, 2008), the median pre-treatment total bilirubin level was higher in the homozygous group (0.80 mg dl^−1^) than in the wild-type and heterozygous groups (both 0.60 mg dl^−1^).

The median administered dose of irinotecan in the first cycle of FOLFIRI was approximately 150 mg m^−2^ in the wild-type and heterozygous groups, as compared with 122.5 mg m^−2^ in the homozygous group, which is nearly 20% less than the recommended dose for FOLFIRI in Japan (150 mg m^−2^) (Muro *et al*, 2010) (Supplementary Table S1). In patients given FOLFIRI, the 3-cycle relative dose-intensities of irinotecan in the homozygous group (55.9%) were lower than those in the wild-type (66.3%) and heterozygous groups (64.1%), irrespective of regimen.

### Safety

**Figure 1:**
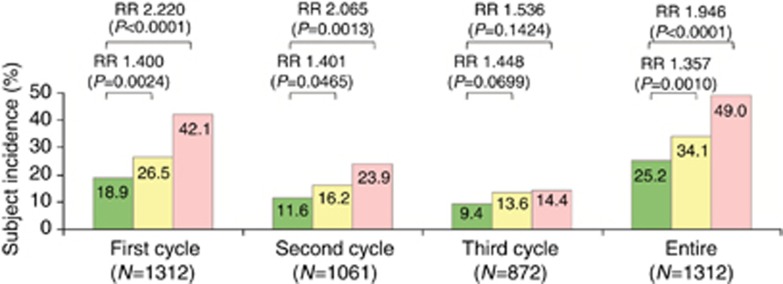
**Subject incidences of grade 3 or 4 neutropenia according to *UGT1A1* genotype.** Green, yellow, and pink bars represent the incidences for patients harbouring *UGT1A1* wild-type (*1/*1), heterozygous (*1/*6, *1/*28), and homozygous (*6/*6, *6/*28, *28/*28) genotypes, respectively. Abbreviation: RR=relative risk.

The incidences of grade 3 or 4 neutropenia in the first cycle and entire treatment cycle (up to the third cycle) were, respectively, 18.9% and 25.2% in the wild-type group, 26.5% and 34.1% in the heterozygous group, and 42.1% and 49.0% in the homozygous group (Figure 1). Severe neutropenia of grade 3 or 4 was more common in the homozygous group (RR, 2.220; *P*<0.0001 in the first cycle; RR, 1.946; *P*<0.0001 in the entire cycle) and heterozygous group (RR, 1.400; *P*=0.0024 in the first cycle; RR, 1.357; *P*=0.0010 in the entire cycle) than in the wild-type group.

Febrile neutropenia developed in 30 (2.3%) of the 1312 eligible patients (11 patients in the wild-type group, 9 in the heterozygous group, and 10 in the homozygous group).

**Figure 2:**
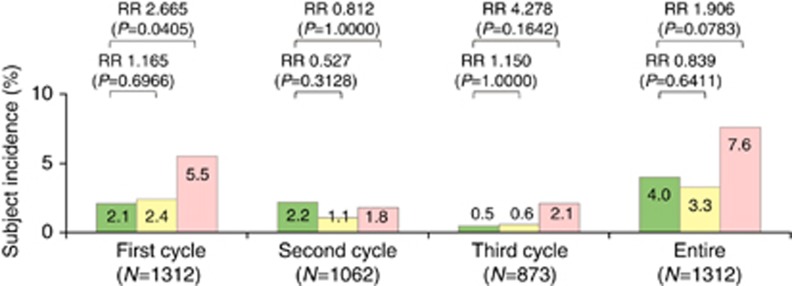
**Subject incidences of grade 3 or 4 diarrhoea according to *UGT1A1* genotype.** Green, yellow, and pink bars represent the incidences for patients harbouring *UGT1A1* wild-type (*1/*1), heterozygous (*1/*6, *1/*28), and homozygous (*6/*6, *6/*28, *28/*28) genotypes, respectively. Abbreviation: RR=relative risk.

The incidences of grade 3 or 4 diarrhoea in the entire cycle were 4.0%, 3.3%, and 7.6% in the wild-type, heterozygous, and homozygous group, respectively (Figure 2). The incidence of severe diarrhoea was significantly higher in the homozygous group (5.5%) than in the wild-type group (2.1%) only in the first cycle (RR, 2.665; *P*=0.0405).

The incidences of severe toxicities in the homozygous group decreased in parallel to the number of treatment cycles (incidences of neutropenia and diarrhoea: 42.1% and 5.5% in first cycle, 23.9% and 1.8% in second cycle, and 14.4% and 2.1% in third cycle, respectively).

### Nomogram development based on the final prediction model and validation

The results of multivariable logistic regression analysis of factors potentially related to severe neutropenia in first cycle of irinotecan-based regimens are shown in Supplementary Table S2. After backward step-down variable selection, treatment line, molecular targeted agents, prior surgery, and prior radiation were excluded from the final prediction model.

The final prediction model for severe neutropenia in the first cycle of irinotecan-based regimens is shown in Table 2. Significant factors (*P*<0.10) included regimen (FOLFIRI *vs* irinotecan+S-1 *vs* irinotecan monotherapy), administered dose of irinotecan, gender (male *vs* female), age, *UGT1A1* genotype (wild-type *vs* heterozygous *vs* homozygous), ECOG PS (0 *vs* 1 *vs*≥2), pre-treatment absolute neutrophil count, and pre-treatment total bilirubin level.

**Figure 3:**
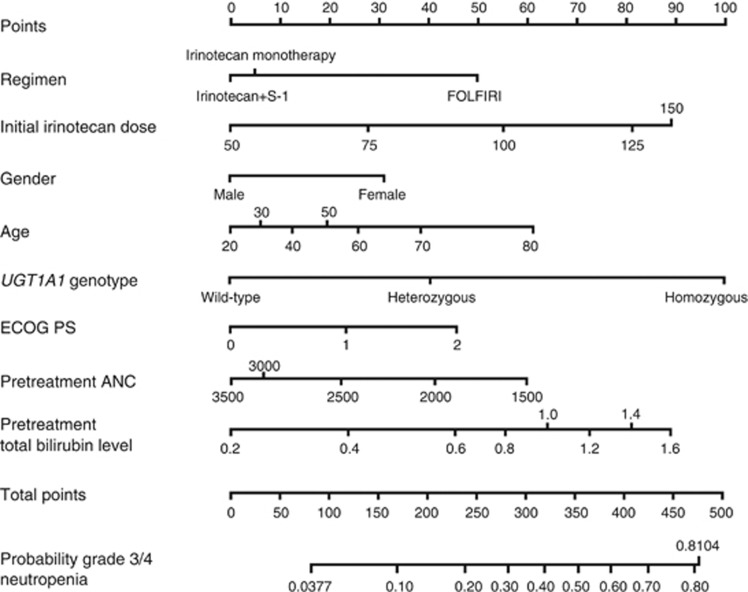
**Nomogram for predicting the probability of irinotecan-induced severe neutropenia in the first cycle.** To calculate the probability of severe (grade 3 or 4) neutropenia, first determine the value for each factor by drawing a vertical line from that factor to the point scale. Then, sum all individual values and draw a vertical line from the total point scale to the probability of severe neutropenia.

**Figure 4:**
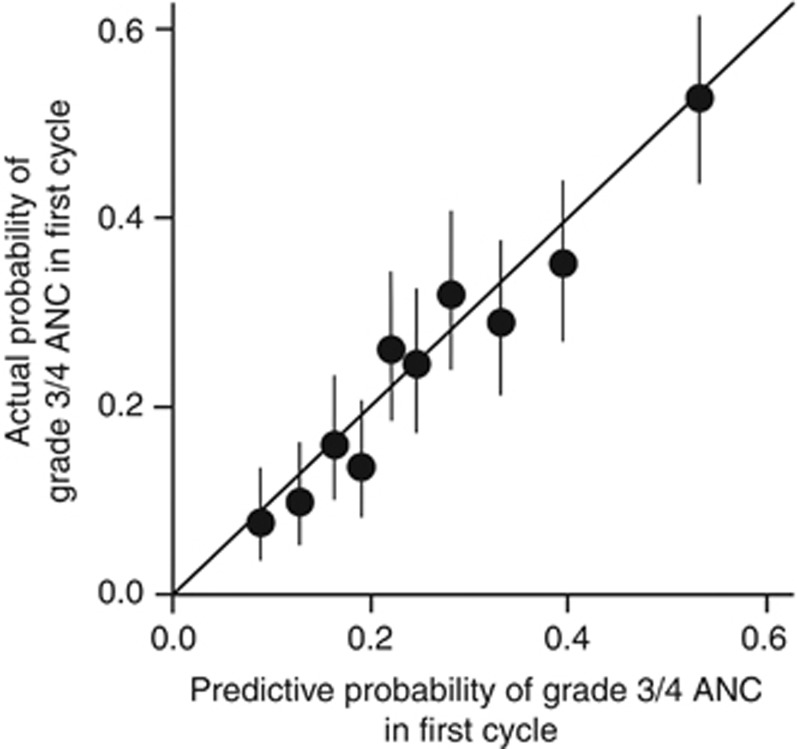
**Nomogram model calibration curve for the internal validation cohort (*n*=1312).** Black line represents ideal fit, where the nomogram-predicted probability (*x*-axis) matches the observed probability (*y*-axis). Closed circles and vertical bars represent the observed probabilities and 95% CI for 10 groups partitioned by the decile of the predicted probabilities. The bootstrap-corrected calibration slope of the regression line in the internal cohort is 1.0026 (95% CI; 0.8053, 1.2038), with an intercept of −0.0005 (95% CI; −0.0485, 0.0428). Abbreviation: CI=confidence interval.

The bootstrap-corrected c-index and the c-index after 10-fold cross-validation were 0.693 and 0.668, respectively. The nomogram for predicting the probability of severe neutropenia during the first cycle of irinotecan-based regimens was internally validated as shown in Figure 3. The bootstrap-corrected calibration slope in the internal cohort is shown in Figure 4, which indicated good agreement between the predicted and observed probabilities of severe neutropenia.

Using data from an independent cohort set (*n*=350), we attempted to validate the nomogram externally. The incidence of severe neutropenia was 25.7% in the external validation cohort. Patients' characteristics were similar in the internal and external validation cohorts, with the exceptions of PS and regimens (Supplementary Table S3). The nomogram also demonstrated good accuracy for predicting the risk of severe neutropenia in the external validation cohort, with a c-index of 0.702. The calibration slope in the external validation cohort was 1.1907, and the intercept −0.0295.

## Discussion

We studied the relation between the safety of irinotecan-based regimens and *UGT1A1* genotype and developed a nomogram to predict the risk of irinotecan-induced severe neutropenia in 1312 advanced colorectal cancer patients registered in a prospective observational study. To our knowledge, our study is the largest prospective study of its type to be performed in Asia; moreover, this is the first nomogram to be validated internally and externally.

Our pre-planned interim analysis of safety confirmed that the *UGT1A1* homozygous and heterozygous genotypes were associated with a higher risk of severe neutropenia than the *UGT1A1* wild-type genotype, consistent with the results of many previous studies (Ando *et al*, 2000; Innocenti *et al*, 2004; Marcuello *et al*, 2004; Rouits *et al*, 2004; Innocenti and Ratain, 2006; Hoskins *et al*, 2007; Minami *et al*, 2007; Kweekel *et al*, 2008; Liu *et al*, 2008; Innocenti *et al*, 2009; McLeod *et al*, 2010; Chen *et al*, 2014). In safety results, regardless of the *UGT1A1* genotype, the subject incidence of severe neutropenia successively decreased in the second and third treatment cycles (Figure 1). This decreasing incidence might have resulted from dose or schedule modifications at the physician's discretion, mainly based on the severity of neutropenia in the previous cycle. Moreover, the homozygous genotype was associated with a two-fold (RR, 2.220) higher risk of severe neutropenia in the first course as compared with wild-type, despite using a lower starting dose in the homozygous group. This finding suggests that the risk of severe neutropenia induced by irinotecan-based regimens cannot be predicted solely on the basis of *UGT1A1* genotype and the administered dose of irinotecan; potential effects of other non-genetic factors such as patients' clinical characteristics must also be considered.

The resulting nomogram demonstrated good accuracy for predicting the probability of severe neutropenia in the first cycle, with a bootstrap-corrected c-index of 0.693 and 0.702 for the internal and external validation cohorts, respectively. The c-index of 0.70 in the external validation cohort indicates that the accuracy of the nomogram for predicting severe neutropenia is 70%, which is considered a clinically meaningful value. Additionally, the *UGT1A1* genotype was the strongest predictor of severe neutropenia among factors included in the final prediction model. In the internal cohort, when only *UGT1A1* genotype was used, the bootstrap-corrected c-index was 0.593 in the prediction model for severe neutropenia in the first cycle of irinotecan-based regimens (odds ratio, 1.545; *P=*0.0020 for heterozygous *vs* wild-type; odds ratio, 3.106; *P*<0.0001 for homozygous *vs* wild-type) (data not shown). The unacceptable discriminative power of the prediction model including only the *UGT1A1* genotype suggests that our comprehensive approach incorporating non-genetic factors provides a more accurate prediction of the risk of severe neutropenia.

A prediction model for severe neutropenia was constructed only for the first cycle, using eight factors chosen by the multivariable logistic regression model (Table 2). We could not construct a prediction model for severe diarrhoea or febrile neutropenia owing to the limited number of patients with such toxicity. Although the total bilirubin level at the start of the first cycle was associated with the *UGT1A1* genotype, both factors were independent predictors. In agreement with our findings, Innocenti *et al* (2009) reported that both the bilirubin level and *UGT1A1* genotype were significant factors in a model including pre-treatment data to predict the risk of severe neutropenia, suggesting that each added predictive value.

Clinically, this internally and externally validated nomogram will most likely be useful for predicting the probability of irinotecan-induced severe neutropenia in patients with colorectal cancer. If the probability of severe neutropenia exceeds the clinically permissible range (e.g., ≥70%), the starting dose of irinotecan should be reduced. The *UGT1A1* genotype-directed dosing of irinotecan has been evaluated in patients receiving irinotecan-based therapy (Toffoli *et al*, 2010; Marcuello *et al*, 2011) or irinotecan monotherapy (Sato *et al*, 2011; Innocenti *et al*, 2014). Patients with a low probability of severe neutropenia (e.g., <30%) as calculated with the nomogram using the standard dose of irinotecan can tolerate substantially higher doses. The concept of nomogram-based dosing of irinotecan should be explored in future clinical trials.

The association between the *UGT1A1* genotype and severe diarrhoea remains controversial (Kweekel *et al*, 2008). Most of the previous studies retrospectively evaluated relatively small numbers of patients with different types of cancer who received various irinotecan-based regimens, although the UGT1A1*28/*28 genotype was associated with a two-fold higher risk of diarrhoea than wild-type genotype in a meta-analysis limited to Caucasians with colorectal cancer (Liu *et al*, 2014). In our study, severe diarrhoea was significantly more common in the homozygous group than in the wild-type group only in the first cycle (RR, 2.665) (Figure 2). The significant association between severe diarrhoea and the *UGT1A1* genotype in our study is attributed to focusing on 1312 patients with advanced colorectal cancer who received three irinotecan-based regimens, supporting that the *UGT1A1* genotype may serve as a predictive marker for irinotecan-induced severe diarrhoea.

Our study had several limitations. First, our results are applicable to only Asians, because the recommended doses and schedules of irinotecan-based regimens differ between Japan and Western countries, S-1 is frequently used in Asia, and the UGT1A1*6 allele is not found in Caucasians. Second, polymorphisms other than UGT1A1*6 or UGT1A1*28, such as *UGT1A7*, *UGT1A9* (Carlini *et al*, 2005; Han *et al*, 2006; Hazama *et al*, 2013), *ABCB1*, *ABCC2*, *ABCG2*, and *SLCO1B1*, have been suggested to be associated with toxicities induced by irinotecan-based regimens (Innocenti *et al*, 2009; Sai *et al*, 2010). Third, our nomogram can only be used to estimate the probability of irinotecan-induced severe neutropenia, but not efficacy. Although several meta-analyses have examined the correlation between *UGT1A1* genotype and the efficacy of irinotecan-based regimens, including tumour response and survival (Palomaki *et al*, 2009; Dias *et al*, 2012; Liu *et al*, 2013; Dias *et al*, 2014), their results remain controversial. The results of our final analysis of outcomes, scheduled to be available in 2015, are expected to shed light on these and other unresolved issues.

In conclusion, our study provides pivotal evidence supporting the association between the *UGT1A1* genotype and an increased risk of irinotecan-induced severe neutropenia and diarrhoea in Japanese patients with colorectal cancer. We developed and validated a clinically useful nomogram including *UGT1A1* genotype and other non-genetic factors for predicting the risk of severe neutropenia in the first cycle of irinotecan-based chemotherapy. We believe that our study represents a great step toward the goal of precision medicine based on irinotecan pharmacogenetics.

## Figures and Tables

**Table 1:** Baseline characteristics

	***UGT1A1*** **genotype group**			
**Characteristics**	**Wild-type (*****N*****=628)**	**Heterozygous (*****N*****=539)**	**Homozygous (*****N*****=145)**	**All (*****N*****=1312)**
**Median age, years (IQR)**	67 (61, 73)	66 (60, 73)	68 (61, 74)	67 (61, 73)
**Gender,** ***n*** **(%)**				
Male	371 (59.1)	362 (67.2)	85 (58.6)	818 (62.3)
Female	257 (40.9)	177 (32.8)	60 (41.4)	494 (37.7)
**Median BSA, m**^**2**^ **(IQR)**	1.52 (1.40, 1.66)	1.57 (1.44, 1.68)	1.54 (1.39, 1.67)	1.54 (1.42, 1.67)
***UGT1A1* genetic profile, *n* (%)**				
Wild-type group				
*1/*1	628 (100.0)			628 (47.9)
Heterozygous group		539 (100.0)		539 (41.1)
*1/*6		308 (57.1)		308 (23.5)
*1/*28		231 (42.9)		231 (17.6)
Homozygous group			145 (100.0)	145 (11.1)
*6/*6			50 (34.5)	50 (3.8)
*28/*28			23 (15.9)	23 (1.8)
*6/*28			72 (49.7)	72 (5.5)
**ECOG PS, *n* (%)**				
0	457 (72.8)	401 (74.4)	109 (75.2)	967 (73.7)
1	142 (22.6)	118 (21.9)	29 (20.0)	289 (22.0)
2	29 (4.6)	20 (3.7)	7 (4.8)	56 (4.3)
**Treatment line, *n* (%)**				
First	138 (22.0)	125 (23.2)	21 (14.5)	284 (21.6)
Second or later	490 (78.0)	414 (76.8)	124 (85.5)	1028 (78.4)
**Regimen, *n* (%)**				
FOLFIRI	395 (62.9)	349 (64.7)	96 (66.2)	840 (64.0)
Irinotecan+S-1	160 (25.5)	132 (24.5)	32 (22.1)	324 (24.7)
Irinotecan monotherapy	73 (11.6)	58 (10.8)	17 (11.7)	148 (11.3)
**Molecular targeted agents, *n* (%)**				
None	236 (37.6)	209 (38.8)	51 (35.2)	496 (37.8)
Anti-VEGF mAb	274 (43.6)	223 (41.4)	64 (44.1)	561 (42.8)
Anti-EGFR mAb	118 (18.8)	107 (19.9)	30 (20.7)	255 (19.4)
**Prior therapy, *n* (%)**				
Surgery	526 (83.8)	456 (84.6)	116 (80.0)	1098 (83.7)
Radiation	46 (7.3)	38 (7.1)	15 (10.3)	99 (7.5)
Chemotherapy	481 (76.6)	417 (77.4)	121 (83.4)	1019 (77.7)
Median WBC, 10^2^ mm^−3^ (IQR)	53.5 (42.6, 66.8)	53.5 (42.0, 69.0)	52.0 (41.3, 63.5)	53.0 (42.0, 67.0)
Median ANC, 10^2^ mm^−3^ (IQR)	31.3 (23.0, 41.7)	31.6 (23.3, 42.5)	31.0 (24.0, 41.0)	31.3 (23.1, 42.0)
Median platelet, 10^4^ mm^−3^ (IQR)	18.7 (14.3, 24.4)	19.1 (14.6, 24.0)	18.3 (14.5, 23.5)	18.8 (14.5, 24.1)
Median total bilirubin level, mg dl^−1^ (IQR)	0.60 (0.40, 0.77)	0.60 (0.50, 0.87)	0.80 (0.60, 1.20)	0.60 (0.46. 0.80)

Abbreviations: ANC=absolute neutrophil count; BSA=body surface area; ECOG PS=Eastern Cooperative Oncology Group performance status; EGFR=epidermal growth factor receptor; FOLFIRI=folinic acid, fluorouracil, and irinotecan; IQR=interquartile range; mAb=monoclonal antibody; *UGT1A1*=uridine diphosphate glucuronosyltransferase 1A1; VEGF=vascular endothelial growth factor; WBC=white blood cells.

**Table 2:** Final prediction model based on multivariable logistic regression analysis for severe neutropenia in the first cycle (*N*=1312)

			**Odds ratio**				
	***N***	***n*** **(%)**	**Estimate**	(**95% CI**)	***P*** **value**	**Overall** ***P***	**c-index**^a^
Regimen							0.693 (0.668)
FOLFIRI	840	241 (28.7)	1	—		0.0015	
Irinotecan+S-1	324	53 (16.4)	0.546	(0.375, 0.794)	0.0015		
Irinotecan monotherapy	148	29 (19.6)	0.579	(0.367, 0.914)	0.0190		
Administered irinotecan dose (mg m^−2^)^b^	1312	323 (24.6)	—	—	—	0.0024	
**Gender**							
Male	818	184 (22.5)	0.686	(0.521, 0.902)	0.0070	0.0070	
Female	494	139 (28.1)	1	—	—		
Age (years)^b^	1312	323 (24.6)	—	—	—	0.0478	
***UGT1A1* genotype**							
Wild-type	628	119 (18.9)	1	—	—	<0.0001	
Heterozygous	539	143 (26.5)	1.624	(1.217, 2.167)	0.0010		
Homozygous	145	61 (42.1)	3.343	(2.191, 5.100)	<0.0001		
**ECOG PS**							
0	967	228 (23.6)	1	—	—	0.0811	
1	289	80 (27.7)	1.330	(0.968, 1.828)	0.0787		
2	56	15 (26.8)	1.749	(0.893, 3.429)	0.1034		
Pre-treatment ANC (mm^−3^)^b^	1312	323 (24.6)	—	—	—	0.0005	
Pre-treatment total bilirubin level, (mg dl^−1^)^b^	1312	323 (24.6)	—	—	—	0.0003	

Abbreviations: ANC=absolute neutrophil count; c-index=concordance index; 95% CI=95% confidence interval; ECOG PS=Eastern Cooperative Oncology Group performance status; FOLFIRI=folinic acid, fluorouracil, and irinotecan; *UGT1A1*=uridine diphosphate glucuronosyltransferase 1A1.

a Bootstrap-corrected c-index (c-index from 10-fold cross-validation).

b Restricted quadratic splines; odds ratios not applicable.

## Notes

WI is a consultant/advisory board member of Daiichi Sankyo and Merck-Serono. KU, KM, YT, HM, SS, MN, AT, and KS report receiving commercial research funds from Daiichi Sankyo. TM reports receiving commercial research funds from Daiichi Sankyo, Taiho Pharmaceutical, and Sanofi. SM is a consultant/advisory board member of Daiichi Sankyo and Taiho Pharmaceutical, and reports receiving commercial research funds from Daiichi Sankyo. YA is a consultant/advisory board member of Daiichi Sankyo, Kowa, GlaxoSmithKline, Novartis, PAREXEL International, and Boehringer Ingelheim, and reports receiving commercial research fund from Novartis. YOku and MS are full-time employees of Daiichi Sankyo. TS and YS are consultant/advisory board members of Daiichi Sankyo. YOha has a leadership role and owns stock of Statcom, is a consultant/advisory board member of Daiichi Sankyo, reports receiving commercial research funds from Astellas Pharma and Takeda Pharmaceutical, and has received travel accommodations from Yakult. No potential conflicts of interest were disclosed by the other authors.

## References

[bib1] Ando Y Saka H Ando M Sawa T Muro K Ueoka H Yokoyama A Saitoh S Shimokata K Hasegawa Y 2000 Polymorphisms of UDP-glucuronosyltransferase gene and irinotecan toxicity: A pharmacogenetic analysis Cancer Res 60 6921 6926 11156391

[bib2] Carlini LE Meropol NJ Bever J Andria ML Hill T Gold P Rogatko A Wang H Blanchard RL 2005 UGT1A7 and UGT1A9 polymorphisms predict response and toxicity in colorectal cancer patients treated with capecitabine/irinotecan Clin Cancer Res 11 1226 1236 15709193

[bib3] Chen YJ Hu F Li CY Fang JM Chu L Zhang X Xu Q 2014 The association of UGT1A1*6 and UGT1A1*28 with irinotecan-induced neutropenia in Asians: a meta-analysis Biomarkers 19 56 62 24308720 10.3109/1354750X.2013.867534

[bib4] Dias MM McKinnon RA Sorich MJ 2012 Impact of the *UGT1A1*28* allele on response to irinotecan: a systematic review and meta-analysis Pharmacogenomics 13 889 899 22676194 10.2217/pgs.12.68

[bib5] Dias MM Pignon J-P Karapetis CS Boige V Glimelius B Kweekel DM Lara PN Laurent-Puig P Martinez-Balibrea E Páez D Punt CJA Redman MW Toffoli G Wadelius M McKinnon RA Sorich MJ 2014 The effect of the UGT1A1*28 allele on survival after irinotecan-based chemotherapy: a collaborative meta-analysis Pharmacogenomics J 14 424 431 24709690 10.1038/tpj.2014.16

[bib6] Goto A Yamada Y Yasui H Kato K Hamaguchi T Muro K Shimada Y Shirao K 2006 Phase II study of combination therapy with S-1 and irinotecan in patients with advanced colorectal cancer Ann Oncol 17 968 973 16603600 10.1093/annonc/mdl066

[bib7] Greenland S 1995 Dose-response and trend analysis in epidemiology: Alternatives to categorical analysis Epidemiology 6 356 365 7548341 10.1097/00001648-199507000-00005

[bib8] Han JY Lim HS Shin ES Yoo YK Park YH Lee JE Jang IJ Lee DH Lee JS 2006 Comprehensive analysis of *UGT1A* polymorphisms predictive for pharmacokinetics and treatment outcome in patients with non-small-cell lung cancer treated with irinotecan and cisplatin J Clin Oncol 24 2237 2244 16636344 10.1200/JCO.2005.03.0239

[bib9] Hazama S Mishima H Tsunedomi R Okuyama Y Kato T Takahashi K Nozawa H Ando H Kobayashi M Takemoto H Nagata N Kanekiyo S Inoue Y Hamamoto Y Fujita Y Hinoda Y Okayama N Oba K Sakamoto J Oka M 2013 *UGT1A1*6*, *1A7*3*, and *1A9*22* genotypes predict severe neutropenia in FOLFIRI-treated metastatic colorectal cancer in two prospective studies in Japan Cancer Sci 104 1662 1669 24033692 10.1111/cas.12283 PMC7653527

[bib10] Hoskins JM Goldberg RM Qu P Ibrahim JG McLeod HL 2007 UGT1A1*28 genotype and irinotecan-induced neutropenia: Dose matters J Natl Cancer Inst 99 1290 1295 17728214 10.1093/jnci/djm115

[bib11] Innocenti F Undevia SD Iyer L Chen PX Das S Kocherginsky M Karrison T Janisch L Ramírez J Rudin CM Vokes EE Ratain MJ 2004 Genetic variants in the *UDP-glucuronosyltranferase 1A1* gene predict the risk of severe neutropenia of irinotecan J Clin Oncol 22 1382 1388 15007088 10.1200/JCO.2004.07.173

[bib12] Innocenti F Ratain MJ 2006 Pharmacogenetics of irinotecan: clinical perspectives on the utility of genotyping Pharmacogenomics 7 1211 1221 17184208 10.2217/14622416.7.8.1211

[bib13] Innocenti F Kroetz DL Schuetz E Dolan ME Ramírez J Relling M Chen P Das S Rosner GL Ratain MJ 2009 Comprehensive pharmacogenetic analysis of irinotecan neutropenia and pharmacokinetics J Clin Oncol 27 2604 2614 19349540 10.1200/JCO.2008.20.6300 PMC2690389

[bib14] Innocenti F Schilsky RL Ramírez J Janisch L Undevia S House LK Das S Wu K Turcich M Marsh R Karrison T Maitland ML Salgia R Ratain MJ 2014 Dose-finding and pharmacokinetic study to optimize the dosing of irinotecan according to the *UGT1A1* genotype of patients with cancer J Clin Oncol 32 2328 2334 24958824 10.1200/JCO.2014.55.2307 PMC4105486

[bib15] Iyer L King CD Whitington PF Green MD Roy SK Tephly TR Coffman BL Ratain MJ 1998 Genetic predisposition to the metabolism of irinotecan (CPT-11). Role of uridine diphosphate glucuronosyltransferase isoform 1A1 in the glucuronidation of its active metabolite (SN-38) in human liver microsomes J Clin Invest 101 847 854 9466980 10.1172/JCI915 PMC508633

[bib16] Komatsu Y Yuki S Sogabe S Fukushima H Iwanaga I Kudo M Tateyama M Meguro T Uebayashi M Saga A Sakata Y Asaka M 2011 Phase II study of combined treatment with irinotecan and S-1 (IRIS) in patients with inoperable or recurrent advanced colorectal cancer (HGCSG0302) Oncology 80 70 75 21659785 10.1159/000328739

[bib17] Kweekel D Guchelaar HJ Gelderblom H 2008 Clinical and pharmacogenetic factors associated with irinotecan toxicity Cancer Treat Rev 34 656 669 18558463 10.1016/j.ctrv.2008.05.002

[bib18] Little RJA Rubin DB 2002 Statistical Analysis with Missing Data (ed 2) Wiley Interscience: Hoboken, NJ

[bib19] Liu CY Chen PM Chiou TJ Liu JH Lin JK Lin TC Chen WS Jiang JK Wang HS Wang WS 2008 UGT1A1*28 polymorphism predicts irinotecan-induced severe toxicities without affecting treatment outcome and survival in patients with metastatic colorectal carcinoma Cancer 112 1932 1940 18300238 10.1002/cncr.23370

[bib20] Liu X Cheng D Kuang Q Liu G Xu W 2013 Association between UGT1A1*28 polymorphisms and clinical outcomes of irinotecan-based chemotherapies in colorectal cancer: A meta-analysis in Caucasians PLoS One 8 e58489 23516488 10.1371/journal.pone.0058489 PMC3597733

[bib21] Liu X Cheng D Kuang Q Liu G Xu W 2014 Association of UGT1A1*28 polymorphisms with irinotecan-induced toxicities in colorectal cancer: a meta-analysis in Caucasians Pharmacogenomics J 14 120 129 23529007 10.1038/tpj.2013.10 PMC3992871

[bib22] Marcuello E Altés A Menoyo A Del Rio E Gómez-Pardo M Baiget M 2004 UGT1A1 gene variations and irinotecan treatment in patients with metastatic colorectal cancer Br J Cancer 91 678 682 15280927 10.1038/sj.bjc.6602042 PMC2364770

[bib23] Marcuello E Páez D Paré L Salazar J Sebio A Del Rio E Baiget M 2011 A genotype-directed phase I-IV dose-finding study of irinotecan in combination with fluorouracil/leucovorin as first-line treatment in advanced colorectal cancer Br J Cancer 105 53 57 21654688 10.1038/bjc.2011.206 PMC3137420

[bib24] McLeod HL Sargent DJ Marsh S Green EM King CR Fuchs CS Ramanathan RK Williamson SK Findlay BP Thibodeau SN Grothey A Morton RF Goldberg RM 2010 Pharmacogenetic predictors of adverse events and response to chemotherapy in metastatic colorectal cancer: Results from North American Gastrointestinal Intergroup Trial N9741 J Clin Oncol 28 3227 3233 20530282 10.1200/JCO.2009.21.7943 PMC2903324

[bib25] Minami H Sai K Saeki M Saito Y Ozawa S Suzuki K Kaniwa N Sawada J Hamaguchi T Yamamoto N Shirao K Yamada Y Ohmatsu H Kubota K Yoshida T Ohtsu A Saijo N 2007 Irinotecan pharmacokinetics/pharmacodynamics and *UGT1A* genetic polymorphisms in Japanese: roles of *UGT1A1*6* and **28* Pharmacogenet Genomics 17 497 504 17558305 10.1097/FPC.0b013e328014341f

[bib26] Muro K Boku N Shimada Y Tsuji A Sameshima S Baba H Satoh T Denda T Ina K Nishina T Yamaguchi K Takiuchi H Esaki T Tokunaga S Kuwano H Komatsu Y Watanabe M Hyodo I Morita S Sugihara K 2010 Irinotecan plus S-1 (IRIS) versus fluorouracil and folinic acid plus irinotecan (FOLFIRI) as second-line chemotherapy for metastatic colorectal cancer: a randomised phase 2/3 non-inferiority study (FIRIS study) Lancet Oncol 11 853 860 20708966 10.1016/S1470-2045(10)70181-9

[bib27] Palomaki GE Bradley LA Douglas MP Kolor K Dotson WD 2009 Can *UGT1A1* genotyping reduce morbidity and mortality in patients with metastatic colorectal cancer treated with irinotecan? An evidence-based review Genet Med 11 21 34 19125129 10.1097/GIM.0b013e31818efd77 PMC2743611

[bib28] Rouits E Boisdron-Celle M Dumont A Guérin O Morel A Gamelin E 2004 Relevance of different UGT1A1 polymorphisms in irinotecan-induced toxicity: A molecular and clinical study of 75 patients Clin Cancer Res 10 5151 5159 15297419 10.1158/1078-0432.CCR-03-0548

[bib29] Sai K Saeki M Saito Y Ozawa S Katori N Jinno H Hasegawa R Kaniwa N Sawada J Komamura K Ueno K Kamakura S Kitakaze M Kitamura Y Kamatani N Minami H Ohtsu A Shirao K Yoshida T Saijo N 2004 *UGT1A1* haplotypes associated with reduced glucuronidation and increased serum bilirubin in irinotecan-administered Japanese patients with cancer Clin Pharmacol Ther 75 501 515 15179405 10.1016/j.clpt.2004.01.010

[bib30] Sai K Saito Y Maekawa K Kim SR Kaniwa N Nishimaki-Mogami T Sawada J Shirao K Hamaguchi T Yamamoto N Kunitoh H Ohe Y Yamada Y Tamura T Yoshida T Matsumura Y Ohtsu A Saijo N Minami H 2010 Additive effects of drug transporter genetic polymorphisms on irinotecan pharmacokinetics/pharmacodynamics in Japanese cancer patients Cancer Chemother Pharmacol 66 95 105 19771428 10.1007/s00280-009-1138-y

[bib31] Saltz L 2013 Systemic therapy for metastatic colorectal cancer J Natl Compr Canc Netw 11 649 652 23704235 10.6004/jnccn.2013.0193

[bib32] Sato T Ura T Yamada Y Yamazaki K Tsujinaka T Munakata M Nishina T Okamura S Esaki T Sasaki Y Koizumi W Kakeji Y Ishizuka N Hyodo I Sakata Y 2011 Genotype-directed, dose-finding study of irinotecan in cancer patients with *UGT1A1*28* and/or *UGT1A1*6* polymorphisms Cancer Sci 102 1868 1873 21740478 10.1111/j.1349-7006.2011.02030.x

[bib33] Senter PD Beam KS Mixan B Wahl AF 2001 Identification and activities of human carboxylesterases for the activation of CPT-11, a clinically approved anticancer drug Bioconjug Chem 12 1074 1080 11716702 10.1021/bc0155420

[bib34] Shimada Y Yoshino M Wakui A Nakao I Futatsuki K Sakata Y Kambe M Taguchi T Ogawa N the CPT-11 Gastrointestinal Cancer Study Group 1993 Phase II study of CPT-11, a new camptothecin derivative, in metastatic colorectal cancer J Clin Oncol 11 909 913 8487053 10.1200/JCO.1993.11.5.909

[bib35] Shiozawa T Tadokoro J Fujiki T Fujino K Kakihata K Masatani S Morita S Gemma A Boku N 2013 Risk factors for severe adverse effects and treatment-related deaths in Japanese patients treated with irinotecan-based chemotherapy: A postmarketing survey Jpn J Clin Oncol 43 483 491 23536639 10.1093/jjco/hyt040 PMC3638635

[bib36] Toffoli G Cecchin E Gasparini G D'Andrea M Azzarello G Basso U Mini E Pessa S De Mattia E Lo ReG Buonadonna A Nobili S De Paoli P Innocenti F 2010 Genotype-driven phase I study of irinotecan administered in combination with fluorouracil/leucovorin in patients with metastatic colorectal cancer J Clin Oncol 28 866 871 20038727 10.1200/JCO.2009.23.6125 PMC4872310

[bib37] Xu G Zhang W Ma MK McLeod HL 2002 Human carboxylesterase 2 is commonly expressed in tumor tissue and is correlated with activation of irinotecan Clin Cancer Res 8 2605 2611 12171891

