# Supplementary material for: An internally and externally validated nomogram for predicting the risk of irinotecan-induced severe neutropenia in advanced colorectal cancer patients
Source: Br J Cancer. 2015 Apr 16;112(10):1709–16. doi: 10.1038/bjc.2015.122 (PMC4430714; doi:10.1038/bjc.2015.122)
Supplement: Supplementary Information [file bjc2015122x1.docx]

Supplementary Table S2. Results of multivariable logistic regression analysis for severe neutropenia in the first cycle (N = 1,312)

|  | | N | n (%) | | Odds ratio | | | |
| --- | --- | --- | --- | --- | --- | --- | --- | --- |
|  |  |  |  |  | Estimate | [95% CI] | *P* value | Overall *P* |
| Treatment line | First | 284 | 66 | (23.2) | 1 | - | - | 0.6031 |
|  | Second or later | 1,028 | 257 | (25.0) | 0.913 | [0.648, 1.286] | 0.6031 |  |
| Regimen | FOLFIRI | 840 | 241 | (28.7) | 1 | - | - | 0.0033 |
|  | Irinotecan + S-1 | 324 | 53 | (16.4) | 0.556 | [0.380, 0.814] | 0.0026 |  |
|  | Irinotecan monotherapy | 148 | 29 | (19.6) | 0.593 | [0.365, 0.964] | .0349 |  |
| Administered irinotecan dose (mg m^-2^)^a^ | | 1,312 | 323 | (24.6) | - | - | - | 0.0022 |
| Gender | Male | 818 | 184 | (22.5) | 0.682 | [0.518, 0.898] | 0.0064 | 0.0064 |
|  | Female | 494 | 139 | (28.1) | 1 | - | - |  |
| Age (years)^a^ | | 1,312 | 323 | (24.6) | - | - | - | 0.0281 |
| *UGT1A1* genotype | Wild-type | 628 | 119 | (18.9) | 1 | - | - | <0.0001 |
|  | Heterozygous | 539 | 143 | (26.5) | 1.635 | [1.224, 2.184] | 0.0009 |  |
|  | Homozygous | 145 | 61 | (42.1) | 3.291 | [2.152, 5.031] | <0.0001 |  |
| ECOG PS | 0 | 967 | 228 | (23.6) | 1 | - | - | 0.1065 |
|  | 1 | 289 | 80 | (27.7) | 1.331 | [0.966, 1.833] | 0.0801 |  |
|  | 2 | 56 | 15 | (26.8) | 1.652 | [0.833, 3.277] | 0.1510 |  |
| Molecular targeted agents | None | 496 | 111 | (22.4) | 1 | - | - | 0.6224 |
|  | Anti VEGF mAb | 561 | 151 | (26.9) | 1.168 | [0.855, 1.595] | 0.3304 |  |
|  | Anti EGFR mAb | 255 | 61 | (23.9) | 1.093 | [0.739, 1.617] | 0.6548 |  |
| Prior surgery | No | 214 | 60 | (28.0) | 1 | - | - | 0.1240 |
|  | Yes | 1098 | 263 | (24.0) | 0.757 | [0.530, 1.080] | 0.1240 |  |
| Prior radiation | No | 1213 | 296 | (24.4) | 1 | - | - | 0.1871 |
|  | Yes | 99 | 27 | (27.3) | 1.401 | [0.849, 2.310] | 0.1871 |  |
| Pretreatment ANC (mm^-3^)^a^ | | 1,312 | 323 | (24.6) | - | - | - | 0.0005 |
| Pretreatment total bilirubin level (mg dL^-1^)^a^ | | 1,312 | 323 | (24.6) | - | - | - | 0.0002 |
| Abbreviation: 95%CI, 95% confidence interval; FOLFIRI, folinic acid, fluorouracil, and irinotecan; UGT1A1, uridine diphosphate glucuronosyltransferase 1A1; ECOG PS, Eastern Cooperative Oncology Group performance status; VEGF, vascular endothelial growth factor; mAb, monoclonal antibody; EGFR, epidermal growth factor receptor; ANC, absolute neutrophil count.  ^a^ Restricted quadratic splines; Odds ratios not applicable. | | | | | | | | |
