# Supplementary material for: An internally and externally validated nomogram for predicting the risk of irinotecan-induced severe neutropenia in advanced colorectal cancer patients
Source: Br J Cancer. 2015 Apr 16;112(10):1709–16. doi: 10.1038/bjc.2015.122 (PMC4430714; doi:10.1038/bjc.2015.122)
Supplement: Supplementary Table S1 [file bjc2015122x2.docx]

Supplementary Table S3. Baseline characteristics of the internal and external validation cohorts (N = 350)

| Characteristics | | Number of patients (%) | |
| --- | --- | --- | --- |
|  |  | Internal cohort  (N = 1,312) | External cohort  (N = 350) |
| **Median age, years (IQR)** | | 67 (61, 73) | 66 (60, 72) |
| **Gender, n (%)** | |  |  |
| Male | | 818 (62.3) | 216 (61.7) |
| Female | | 494 (37.7) | 134 (38.3) |
| **Median BSA, m^2^ (IQR)** | | 1.54 (1.42, 1.67) | 1.57 (1.49, 1.65) |
| ***UGT1A1* genetic profile, n (%)** | |  |  |
| Wild-type group | **1/*1* | 628 (47.9) | 180 (51.4) |
| Heterozygous group |  | 539 (41.1) | 133 (38.0) |
|  | **1/*6* | 308 (23.5) | 85 (24.3) |
|  | **1/*28* | 231 (17.6) | 48 (13.7) |
| Homozygous group |  | 145 (11.1) | 37 (10.6) |
|  | **6/*6* | 50 (3.8) | 20 (5.7) |
|  | **28/*28* | 23 (1.8) | 7 (2.0) |
|  | **6/*28* | 72 (5.5) | 10 (2.9) |
| **ECOG PS, n (%)** | |  |  |
| 0 | | 967 (73.7) | 208 (59.4) |
| 1 | | 289 (22.0) | 130 (37.1) |
| 2 | | 56 (4.3) | 12 (3.4) |

| **Treatment line, n (%)** |  |  |
| --- | --- | --- |
| First | 284 (21.6) | 155 (44.3) |
| Second or later | 1,028 (78.4) | 195 (55.7) |
| **Regimen, n (%)** |  |  |
| FOLFIRI | 840 (64.0) | 222 (63.4) |
| Irinotecan + S-1 | 324 (24.7) | 31 (8.9) |
| Irinotecan monotherapy | 148 (11.3) | 97 (27.7) |
| **Median WBC, 10^2^ mm^-3^ (IQR)** | 53.0 (42.0, 67.0) | 56.0 (44.0, 71.0) |
| **Median ANC, 10^2^ mm^-3^ (IQR)** | 31.3 (23.1, 42.0) | 35.3 (26.1, 47.0) |
| **Median platelet, 10^4^ mm^-3^ (IQR)** | 18.8 (14.5, 24.1) | 19.9 (17.1, 26.3) |
| **Median total bilirubin level, mg dL^-1^ (IQR)** | 0.60 (0.46. 0.80) | 0.60 (0.40, 0.80) |
| Abbreviation: IQR, interquartile range; BSA, body surface area; UGT1A1, uridine diphosphate glucuronosyltransferase 1A1; ECOG PS, Eastern Cooperative Oncology Group performance status; FOLFIRI, folinic acid, fluorouracil, and irinotecan; WBC, white blood cells; ANC, absolute neutrophil count. | | |
