# Supplementary material for: An internally and externally validated nomogram for predicting the risk of irinotecan-induced severe neutropenia in advanced colorectal cancer patients
Source: Br J Cancer. 2015 Apr 16;112(10):1709–16. doi: 10.1038/bjc.2015.122 (PMC4430714; doi:10.1038/bjc.2015.122)
Supplement: Supplementary Table S2 [file bjc2015122x3.docx]

Appendix

Recruiting institutions (site principal investigators) contributing with at least one patient onto this study were as follows:

Nagoya University Graduate School of Medicine (Keisuke Uehara), Mitsui Memorial Hospital (Keisuke Minamimura), Gifu Prefectural General Medical Centre (Katsuyuki Kunieda), Niigata Cancer Centre Hospital (Yasumasa Takii), Chiba University Graduate School of Medicine (Hideaki Miyauchi), Tokai University School of Medicine (Sotaro Sadahiro), Nishijin Hospital (Kanehisa Fukumoto), Hiroshima Prefectural Hospital (Katsunori Shinozaki), Chugoku Central Hospital (Takeshi Kambara), Dongo Hospital (Toshio Otsuji), Graduate School of Medical Sciences, Kumamoto University (Hideo Baba), Osaki Citizen Hospital (Makio Gamoh), Matsue Red Cross Hospital (Kenji Takubo), Fukui-Ken Saiseikai Hospital (Yoshinori Munemoto), Hokkaido PWFAC Asahikawa-Kosei General Hospital (Shiro Nakano), National Hospital Organization, Higashihiroshima Medical Centre (Tadateru Takahashi), Hiroshima City Asa Hospital (Naoki Hirabayashi), Kyorin University School of Medicine (Tadahiko Masaki), Rakuwakai-Otowa Hospital (Ryoji Takeda), Japanese Red Cross Society, Wakayama Medical Centre (Shiro Uyama), Keiaido Hospital (Tsuyoshi Arai), Ishikawa Prefectural Central Hospital (Hiroyuki Bando), Toyohashi Municipal Hospital (Kazuhiro Hiramatsu), Akita University Graduate School of Medicine (Hideaki Miyazawa), Akita Red Cross Hospital (Osamu Muto), Saiseikai Futsukaichi Hospital (Kazuya Naritomi), Izumi Municipal Hospital (Ryugo Sawada), Kanagawa Cancer Centre (Manabu Shiozawa), Yokkaichi Municipal Hospital (Hitoshi Teramoto), Torii Clinic (Tsuyoshi Torii), Akishima Hospital (Atsushi Uehara), Hirakata Kohsai Hospital (Takataro Fukuhara), Hokkaido PWFAC Sapporo-Kosei General Hospital (Hiroshi Kozawa), Matsue City Hospital (Masahiko Miura), Daini Okamoto General Hospital (Yoshihiro Shimizu), Konan Hospital (Satoshi Tani), Koga General Hospital (Shoji Taniguchi), Meijo Hospital (Toshio Uematsu), Masuda Medical Association Hospital (Masahiko Igarashi), Tohoku University Hospital (Chikashi Ishioka), Kitakyushu Municipal Yahata Hospital (Shigehiko Ito), Kochi Medical School Hospital (Michiya Kobayashi), Mutsu General Hospital (Osamu Matsuura), University of Occupational and Environmental Health (Koji Yamaguchi), Shiga Medical Centre (Masazumi Zaima), Saiseikai Arida Hospital (Hiromitsu Fukunaga), Nagasaki Goto Chuoh Hospital (Junichiro Furui), Teikyo University Chiba Medical Centre (Keiji Koda), Hamamatsu University School of Medicine (Kiyotaka Kurachi), Yokohama Ekisaikai Hospital (Yasuhiko Miura), Kakogawa West-City Hospital (Tetsuya Sakai), Juntendo University Faculty of Medicine (Kazuhiro Sakamoto), Tokyo Teishin Hospital (Kanetoshi Sato), Osaka-kita Teishin Hospital (Yukihiko Tokunaga), Yodogawa Christian Hospital (Akihiro Toyokawa), Gunma University Graduate School of Medicine (Soichi Tsutsumi), Aichi Prefectural Welfare Federation of Agricultural Cooperatives Kainan Hospital (Toyohisa Yaguchi), Oita Red Cross Hospital (Kengo Fukuzawa), Nishisaitama-chuo National Hospital (Takenori Hayashi), Shimane University Faculty of Medicine (Noriyuki Hirahara), Kyoto Prefectural University of Medicine Graduate School of Medical Science (Takeshi Ishikawa), Keiju Medical Centre (Tooru Kamata), Kochi Health Sciences Centre (Yuji Negoro), St. Luke's International Hospital (Keiichiro Ohta), Social Insurance Tagawa Hospital (Tomoya Sudo), Anan Kyoei Hospital (Michio Ando), Naga Hospital (Kazuo Arii), Tottori University Hospital (Keigo Ashida), Hakuai Hospital (Masayuki Ataka), Ryugasaki Saiseikai Hospital (Tsugio Ebihara), Kagawa University Hospital (Fuminori Goda), Japanese Red Cross Nagoya Daini Hospital (Hiroshi Hasegawa), Teikyo University School of Medicine (Yojiro Hashiguchi), Gunma-ken Saiseikai Maebashi Hospital (Yasuo Hosouchi), Hamamatsu Rosai Hospital (Harutaka Inoue), Saiseikai Saijo Hospital (Hiroshi Ishii), Tatebayashi Kosei Hospital (Shigeru Iwazaki), Senseki Hospital (Mariko Kambe), Kobe City Hospital Organization Kobe City Centre West Hospital (Yuichiro Kikawa), Miyazaki University School of Medicine (Kazuo Kitamura), Hokkaido University Hospital (Yoshito Komatsu), Kagoshima Prefectural Kanoya Medical Centre (Masaaki Kubo), Nagano Prefectural Kiso Hospital (Shigeyoshi Kumeda), Yokohama City University Medical Centre (Chikara Kunisaki), Nishikobe Medical Centre (Takahisa Kyogoku), Hiroshima Red Cross Hospital and Atomic-bomb Survivors Hospital (Hiroyuki Matsuda), Tsukuba Memorial Hospital (So Matsui), Shiga University of Medical Science (Eiji Mekata), Miyagi Cancer Centre (Yasuko Murakawa), Okayama University Graduate School of Medicine, Dentistry and Pharmaceutical Sciences (Takeshi Nagasaka), Saiseikai Nakatsu Hospital (Shiro Nakae), Japanese Red Cross Kanazawa Hospital (Genichi Nishimura), Saiseikai Yawata General Hospital (Kenichi Nomoto), Aichi Medical University School of Medicine (Toshiaki Nonami), Mie Prefectural General Medical Centre (Eiki Ojima), Fukui National Hospital (Hidetoshi Onchi), Nagoya City University Graduate School of Medical Sciences (Mikinori Satoh), Nagano Red Cross Hospital (Harutsugu Sodeyama), Yamaguchi Grand Medical Centre (Ryuichiro Sudo), Sera Central Hospital (Shinichi Suehiro), Graduate School of Medicine and Pharmaceutical Sciences University of Toyama (Toshiro Sugiyama), Yamanashi Red Cross Hospital (Yusuke Tajima), Shunan Memorial Hospital (Motohiro Takeshige), Chukyo Hospital (Yuichiro Tojima), Higashiomiya General Hospital (Atsushi Umemoto), Juntendo University Faculty of Medicine (Sumio Watanabe), Fukuoka University School of Medicine (Yuichi Yamashita), Meiwa Hospital (Hidenori Yanagi), National Hospital Organization Takasaki General Medical Centre (Masaaki Aiba), Akita City Hospital (Suguru Hasegawa), Yokohama City University Graduate School of Medicine (Yasushi Ichikawa), JA Hiroshima General Hospital (Yuji Imamura), Fujiyoshida Municipal Hospital (Hitoshi Ishikawa), Yamaguchi Rosai Hospital (Tomoe Kato), Bell-land General Hospital (Masayasu Kawasaki), Wakayama Rosai Hospital (Yasuhito Kobayashi), Nippon Medical School (Satoshi Matsumoto), Kureha General Hospital (Yasuhiko Midorikawa), Fukuoka University Chikushi Hospital (Koji Mikami), Kagoshima Prefectural Satsunan Hospital (Shinji Mitsue), Okinawa Chubu Hospital (Takahiro Murakami), Saint Martin's Hospital (Takayoshi Murakami), Imakiire General Hospital (Hiroshi Mure), Okinawa Prefectural Nanbu Medical Centre and Children's Medical Centre (Naoji Nagamine), Miyazaki Prefectural Nobeoka Hospital (Tetsufumi Ohchi), Saiseikai Kawaguchi General Hospital (Masahiko Sato), Shinrakuen Hospital (Osamu Sato), Yokohama Sakae Kyosai Hospital (Hiro Satoh), The University of Tokushima Graduate School (Mitsuo Shimada), Kagawa Prefectural Central Hospital (Ichio Suzuka), Tottori-Seikyo Hospital (Kazunori Suzuki), The University of Tokushima Graduate School (Tetsuji Takayama), Minamata City General Hospital and Medical Centre (Tomio Tanigawa), Miyoshi Central Hospital (Naokuni Tatsumoto), NTT East Japan Tohoku Hospital (Takayuki Terasawa), Hiraka General Hospital (Shigeki Tsukamoto), Okayama University Graduate School of Medicine, Dentistry and Pharmaceutical Sciences (Kazunori Tsukuda), Tokyo Medical and Dental University (Hiroyuki Uetake), Ishikiri-Seiki Hospital (Takatsugu Yamamoto), Kitakyushu Municipal Medical Centre (Hirotada Akiho), National Hospital Organization Oita Medical Centre (Hideaki Anai), Fukui Prefectural Hospital (Kenji Doden), Nippon Medical School (Akihito Ehara), Shiga University of Medical Science (Yoshihiro Endo), Kohsei Chuo General Hospital (Masanobu Enomoto), Jiaikai Imamura Hospital (Shuichi Hokita), Terada Hospital (Takashi Hori), Tokorozawa Central Hospital (Jun Imai), Nagoya Memorial Hospital (Kenji Ina), Mie University Graduate School of Medicine (Yasuhiro Inoue), Yaizu City Hospital (Yukio Ishihara), Saiseikai Hiroshima Hospital (Akira Kameda), Kobe Kaisei Hospital (Kunihiko Kaneda), Kanazawa Medical University (Takeo Kosaka), Akashi Medical Centre (Hirofumi Kosuga), Dokkyo Medical University (Keiichi Kubota), Miki City Hospital (Takashi Munezane), Musashino Red Cross Hospital (Minoru Nakane), Kyoto Prefectural University of Medicine Graduate School of Medical Science (Masayoshi Nakanishi), Tsukuba-Gakuen Hospital (Masaaki Nishi), University of the Ryukyus Graduate School of Medicine (Tadashi Nishimaki), Japanese Red Cross Nagoya Daiichi Hospital (Fumio Nomura), Hiroshima City Hiroshima Citizens Hospital (Yasutomo Ojima), Yashima General Hospital (Makoto Saito), Yatsuo General Hospital (Mitsukazu Saito), Hirosaki University Graduate School of Medicine (Atsushi Sato), Maki Hospital (Mutsuya Sato), Saiseikai Wakakusa Hospital (Yasuo Sato), Akita University Graduate School of Medicine (Hiroyuki Shibata), Takamatsu Red Cross Hospital (Mitsushige Shibatoge), Saiseikai Toyama Hospital (Tetsuro Shimizu), Hokkaido University Hospital (Yasushi Shimizu), Chigasaki Municipal Hospital (Hiroshi Shinkai), Takayama Red Cross Hospital (Takashi Shiroko), South Miyagi Medical Centre (Katsuo Sugiyama), Yokohama Shin-midori General Hospital (Hiroki Sumitomo), Kouseiren Namerikawa Hospital (Shigehiro Tanaka), Asahikawa City Hospital (Masaki Taruishi), Haramachi Red Cross Hospital (Nobuyuki Uchida), Mitoyo General Hospital (Tetsunobu Udaka), Noto General Hospital (Satoshi Ushijima), Isahaya General Hospital (Hiroyuki Yamaguchi), Hidaka General Hospital (Kazuya Yamaguchi), Kitamurayama Hospital (Kazuhito Yamamoto), National Hospital Organization, Okayama Medical Centre (Haruhiro Yamashita), Saiseikai Nakatsu Hospital (Hiroshi Yamashita), Wakayama Medical University (Hiroki Yamaue), Japan Community Health Care Organization Kanazawa Hospital (Toshiaki Yasui), Gifu University Graduate School of Medicine (Kazuhiro Yoshida), Nagoya Ekisaikai Hospital (Atsushi Akutagawa), Kyorin University School of Medicine (Junji Furuse), National Hospital Organization Kanmon Medical Centre (Takumi Furuya), Kagoshima City Hospital (Nobuo Hamada), Tarumizu Central Hospital (Shigeya Hase), Oita Oka Hospital (Yoshinori Hirashima), Tsukuba Memorial Hospital (Kazuto Ikezawa), Hokkaido PWFAC Engaru-Kosei General Hospital (Satoshi Inaba), National Hospital Organization Shikoku Medical Centre for Children and Adults (Aiichiro Kajikawa), Midori Municipal Hospital (Yasuhiro Kamiya), Nagayoshi General Hospital (Susumu Kaseno), Aichi Medical University School of Medicine (Kunio Kasugai), Institute of Biomedical Research and Innovation (Nobuyuki Katakami), National Hospital Organization Yonago Medical Centre (Takuji Naka), Kawaguchi Seiwa Hospital (Jun Kinoshita), Seirei Mikatahara General Hospital (Yukihiro Kunimoto), Japanese Red Cross Nagahama Hospital (Kazuhiro Maruhashi), Yokohama Minami Kyousai Hospital (Hiroshi Matsukawa), Aomori Prefectural Central Hospital (Masaki Munakata), Mabi Memorial Hospital (Tomoyoshi Muramatsu), Saiseikai Matsusaka General Hospital (Tatsushi Naganuma), Yamatokashihara Hospital (Mitsuo Nagao), Kanazawa University Graduate School of Medicine (Keishi Nakamura), Seirei Numazu Hospital (Hironari Nasuno), Sapporo City General Hospital (Shuji Nishikawa), Shizugawa Public Hospital (Masafumi Nishizawa), Shizuoka General Hospital (Ko Ohata), Hiroshima University Graduate School of Biomedical Sciences (Hideki Ohdan), Konan Kakogawa Hospital (Kyosuke Ohta), Hiroshima Kyoritsu Hospital (Sunao Ohtagaki), Saiseikai Ohmuta Hospital (Takafumi Ono), Saiseikai Niigata Daini Hospital (Yasuo Sakai), Nagoya City West Medical Centre (Kenichi Sakakibara), Hakui General Hospital (Noriaki Sakata), Shiso Municipal Hospital (Shinsuke Satake), Juntendo Shizuoka Hospital, Juntendo University School of Medicine (Koichi Sato), Keiyu Hospital (Akihiko Shimada), Nippon Dental University (Takeyasu Suda), Saiseikai Yamaguchi General Hospital (Tsuyoshi Takahashi), Shimaneken Saiseikai Gotsu General Hospital (Masataka Takebayashi), Fukuoka University School of Medicine (Kazuo Tamura), Hikone Municipal Hospital (Yasufumi Teramura), Shimane Prefectural Central Hospital (Atsuo Tokuka), Tosei General Hospital (Kenji Tsuboi), Takagi Hospital (Masashi Tsugita), Japanese Red Cross Okayama Hospital (Tetsuya Tsurumi), Japanese Red Cross Society Himeji Hospital (Takanori Watanabe), Saiseikai Kyoto Hospital (Masaharu Yabe), Tokushima Prefectural Central Hospital (Yoshiyuki Yagi), Kokuho Central Hospital (Yukishige Yamada), Kanagawa Prefectural Ashigarakami Hospital (Yuji Yamamoto), Rakusai Newtown Hospital (Nobuki Yamaoka), Otsu Municipal Hospital (Ken Yanagibashi), Tenri Hospital (Tsunehiro Yoshimura), NTT East Corporation Sapporo Hospital (Nobuaki Akakura), Kamiamakusa General Hospital (Makoto Fukuda), Numazu City Hospital (Toru Fukunaga), Tsukuba-Gakuen Hospital (Takeshi Gohongi), Yame General Hospital (Mamoru Hiraki), Tonami General Hospital (Kenichi Ietsugu), Nishimino Kosei Hospital (Tatsumi Iida), JA Hiroshima General Hospital (Kunio Ishida), Toranomon Hospital (Mitsuru Kaise), Kusunoki Hospital (Masashi Kamio), Hasuda Hospital (Jun Kaneko), Taragi Municipal Hospital (Hiroshi Kaneta), Musashino Red Cross Hospital (Yasuyuki Kawachi), Nagoya University Graduate School of Medicine (Yasuhiro Kodera), Asahikawa Red Cross Hospital (Kyuhei Kohda), Kohdaira Hospital (Fujio Kohdaira), Tone Central Hospital (Takayuki Kohri), Saiseikai Kurihashi Hospital (Taro Koike), Miyazaki University School of Medicine (Kazuhiro Kondo), Otaru Municipal Hospital (Yoshihiro Kondo), Yamanashi Prefectural Central Hospital (Yoshiaki Miyasaka), Shimotsuga General Hospital (Naoki Morimoto), Nagano Municipal Hospital (Yasuhiro Munakata), Nakashibetsu Town Hospital (Tomonori Nakagawa), JA Onomichi General Hospital (Masahiro Nakahara), Houju Memorial Hospital (Hideo Nakajima), Kyushu Rosai Hospital (Hiroshi Nakashima), Kagoshima University Graduate School of Medical and Dental Sciences (Shoji Natsugoe), Oita Sanai Medical Centre (Fumihiko Niiya), Medical City Tobu Hospital (Yoshikazu Ohta), Niigata Prefectural Central Hospital (Takayuki Okada), Kitano Hospital (Nobuhiro Osaki), National Hospital Organization Yokohama Medical Centre (Hitoshi Sekido), Hiroshima University Graduate School of Biomedical Sciences (Tadashi Senoo), JA Yoshida General Hospital (Naofumi Shigeta), Yoka Hospital (Akira Sugezawa), Kensei General Hospital (Hitoshi Takahashi), Public Central Hospital of Matto Ishikawa (Toshiya Takeda), Ina Central Hospital (Nobumichi Takeuchi), Sakai Hospital, Kinki University School of Medicine (Akira Tanaka), Tomakomai Nissho Hospital (Miki Tateyama), Iwate Prefectural Kamaishi Hospital (Chihiro Tono), Sakado Central Hospital (Choji Tsuchiya), National Hospital Organization Himeji Medical Centre (Yasuo Wada), Mimihara General Hospital (Takuya Yamaguchi), Kawasaki Medical School (Yoshiyuki Yamaguchi), Mizushima Kyodo Hospital (Akihiro Yamamoto), Tokushima Prefectural Central Hospital (Mitsuyasu Yano), Chiba University Graduate School of Medicine (Hiroyuki Yoshidome), Yamagata University Faculty of Medicine (Takashi Yoshioka).
