# Supplementary material for: An internally and externally validated nomogram for predicting the risk of irinotecan-induced severe neutropenia in advanced colorectal cancer patients
Source: Br J Cancer. 2015 Apr 16;112(10):1709–16. doi: 10.1038/bjc.2015.122 (PMC4430714; doi:10.1038/bjc.2015.122)
Supplement: Supplementary Table S3 [file bjc2015122x4.docx]

Supplementary Table S1. Administered dose in the first cycle and 3-cycle relative dose-intensity^a^ of irinotecan

|  |  | | *UGT1A1* genotype group | | | |
| --- | --- | --- | --- | --- | --- | --- |
|  |  | | Wild-type | Heterozygous | Homozygous | All |
| FOLFIRI | N | | 395 | 349 | 96 | 840 |
|  | Administered dose in the first cycle | Actual dose (mg m^-2^),  median (IQR) | 146.9  (123.1, 151.7) | 145.2  (121.4, 150.9) | 122.5  (98.8, 149.7) | 144.9  (120.0, 151.0) |
|  |  | Percentage of standard dose (%), median (IQR) | 98.0  (82.1, 101.1) | 96.8  (80.9, 100.6) | 81.7  (65.8, 99.8) | 96.6  (80.0, 100.7) |
|  | 3-cycle RDI (%), median (IQR) | | 66.3  (50.1, 82.9) | 64.1  (49.6, 80.7) | 55.9  (41.4, 68.4) | 64.2  (49.1, 80.8) |
| Irinotecan + S-1 | N | | 160 | 132 | 32 | 324 |
|  | Administered dose in the first cycle | Actual dose (mg m^-2^), median (IQR) | 103.9  (91.3, 125.0) | 100.9  (80.8, 124.0) | 99.8  (75.0, 108.0) | 101.4  (83.4, 124.6) |
|  |  | Percentage of standard dose (%), median (IQR) | 81.5  (71.8, 99.9) | 80.0  (64.4, 98.6) | 68.2  (60.0, 80.7) | 80.2  (65.9, 98.7) |
|  | 3-cycle RDI (%), median (IQR) | | 65.7  (50.3, 82.7) | 60.2  (46.7, 75.7) | 53.3  (40.1, 60.6) | 60.6  (48.8, 79.5) |
| Irinotecan monotherapy | N | | 73 | 58 | 17 | 148 |
|  | Administered dose in the first cycle | Actual dose (mg m^-2^), median (IQR) | 132.1  (104.6, 149.3) | 116.6  (99.1, 150.0) | 100.0  (74.8, 121.5) | 121.3  (99.5, 149.2) |
|  |  | Percentage of standard dose (%), median (IQR) | 98.1  (82.2, 101.0) | 81.8  (66.4, 100.0) | 66.7  (55.4, 81.0) | 91.4  (68.0, 100.0) |
|  | 3-cycle RDI (%), median (IQR) | | 67.7  (49.4, 98.9) | 65.4  (49.3, 87.4) | 45.0  (39.9, 72.2) | 64.9  (47.0, 91.4) |
| ^a^ 3-cycle relative dose-intensities were calculated as the delivered dose-intensity divided by literature-derived typical dose-intensity in Japanese clinical trials.  Abbreviation: UGT1A1, uridine diphosphate glucuronosyltransferase 1A1; FOLFIRI, folinic acid, fluorouracil, and irinotecan; IQR, interquartile range; RDI, relative dose-intensity. | | | | | | |
